# An Update on the Effects of Glyceollins on Human Health: Possible Anticancer Effects and Underlying Mechanisms

**DOI:** 10.3390/nu11010079

**Published:** 2019-01-03

**Authors:** Thu Ha Pham, Sylvain Lecomte, Theo Efstathiou, Francois Ferriere, Farzad Pakdel

**Affiliations:** 1 Univ Rennes, Inserm, EHESP, Irset (Institut de recherche en santé, environnement et travail)—UMR_S 1085, F-35000 Rennes, France; thu-ha.pham@univ-rennes1.fr (T.H.P.); sylvain.lecomte35@gmail.com (S.L.); francois.ferriere@univ-rennes1.fr (F.F.); 2 Laboratoire Nutrinov, Technopole Atalante Champeaux, 8 rue Jules Maillard de la Gournerie, 35012 Rennes Cedex, France; theo.efstathiou@nutrinov.com

**Keywords:** glyceollins, phytochemicals, dietary compounds, signaling pathways, estrogen receptor, breast cancer, human health

## Abstract

Biologically active plant-based compounds, commonly referred to as phytochemicals, can influence the expression and function of various receptors and transcription factors or signaling pathways that play vital roles in cellular functions and are then involved in human health and diseases. Thus, phytochemicals may have a great potential to prevent and treat chronic diseases. Glyceollins, a group of phytoalexins that are isolated from soybeans, have attracted attention because they exert numerous effects on human functions and diseases, notably anticancer effects. In this review, we have presented an update on the effects of glyceollins in relation to their potential beneficial roles in human health. Despite a growing number of studies suggesting that this new family of phytochemicals can be involved in critical cellular pathways, such as estrogen receptor, protein kinase, and lipid kinase signaling pathways, future investigations will be needed to better understand their molecular mechanisms and their specific significance in biomedical applications.

## 1. Introduction

Thousands of bioactive molecules, among phytochemicals, naturally occur in plants after secondary metabolism. Out of these compounds, polyphenols represent the most important family, including more than 500 identified compounds [1,2]. They are mainly composed of flavonoids, phenolic acids, stilbenes, and lignans, found in a wide variety of plant-based foods. Polyphenol concentrations in foods and quantities consumed differ considerably, leading to wide differences in the daily intake between polyphenol compounds [2]. Polyphenols have often been associated with a decreased incidence of several human diseases and have been used in traditional medicines and functional foods [3]. Epidemiological studies have shown that a high intake of polyphenolic phytochemicals in the diet can prevent many diseases, such as cancers, diabetes or inflammatory, cardiovascular, and neurodegenerative diseases [4,5,6,7,8,9]. However, these studies have some limitations because they do not always take into account the geographical diversity, sex, age, and region of residence of the subjects. In addition, an accurate evaluation of polyphenol intake, which appears to be essential in these studies, is not always completed [2,8]. Polyphenols are also used as food supplements and in pharmaceutical and cosmetic products [10,11,12]. One of the advantages of ingesting phytochemicals through the diet is that combinatory effects from these compounds can occur [9]. 

The phytoalexins, a group of polyphenolic compounds possessing strong antimicrobial and antifungal properties, are produced by plants as defense molecules against phytopathogens [13]. They are only present in very small quantities in healthy plants, but accumulate in large quantities following an attack by bacteria, fungi, or nematodes [14,15]. In addition to biotic agents, other stresses, such as high temperature, ultraviolet (UV) radiation, humidity, and dryness, can also induce the production of these compounds [14,15]. Phytoalexins are very diverse and have been found in various crops, such as rice [16], soybean [17], maize [18], barley [19], and banana [20]. Phytoalexins can be produced in numerous parts of the plant, such as the flowers, leaves, stems, seeds, and root tubers [19,21].

**Figure 1:**
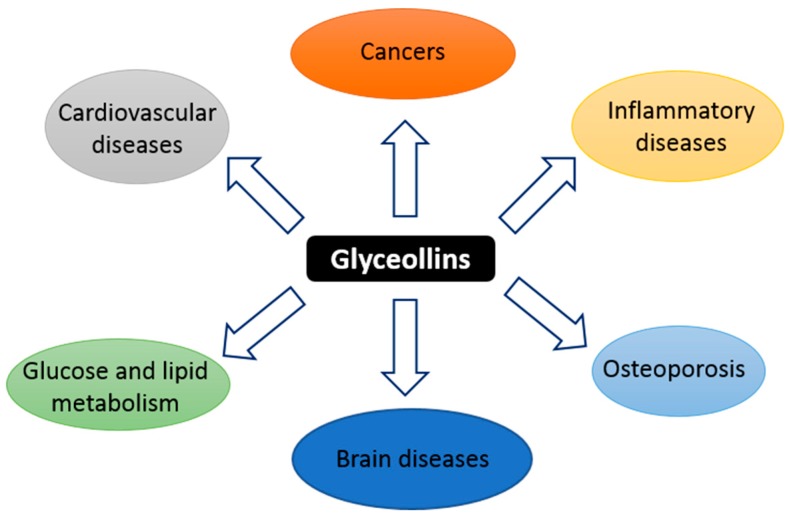
Schematic summary of the targets of glyceollins.

Glyceollins, a group of phytoalexins, are the most important bioactive compounds present in soybeans upon exposure to certain fungi and some abiotic elicitors, such as UV light, aluminum chloride, or methyl jasmonate [17,22]. Glyceollin I, II, and III (GI, GII, and GIII) are de novo synthesized from the soy isoflavone daidzein. In addition to their antibacterial, antifungal, and antinematode actions, glyceollins have recently received much attention because of their antiproliferative, antiestrogenic, anti-inflammatory, antioxidative, and anticholesterolemic activities. They may possess potential medicinal properties in humans, notably protective effects against hormone-dependent cancers, and metabolic and cardiac diseases (Figure 1) [23,24]. Accordingly, glyceollins are effectively able to inhibit proinflammatory cytokines by inhibiting the activation/phosphorylation of the transcription nuclear factor-kappa B (NF-κB). They prevented the lipopolysaccharide (LPS)-induced expression of nitric oxide synthase (iNOS) and cyclo-oxygenase (COX)-2 in murine macrophage cell lines and were able to reduce 12-*O*-tetradecanoylphorbol-13-acetate (TPA)-induced skin inflammation in mice. These natural compounds also inhibit hormone-dependent breast and ovarian cancer cell growth [25,26]. One of the mechanisms responsible for the antitumoral effect of glyceollins is their antiestrogenic action [25,27,28]. However, other antitumorigenic actions, whose mechanisms are not well understood, of glyceollins have also been reported. For instance, Lee et al. reported on an inhibitory effect of glyceollins on the kinase activity of the vascular endothelial growth factor (VEGF) receptor as well as its downstream signal transduction pathways involved in angiogenesis and tumor growth [29]. Likewise, an inhibitory effect on the expression of hypoxia inducible factor 1 (HIF-1)-target genes (such as VEGF) in cancer cells was described [30]. In addition, glyceollins were able to inhibit phosphatidylinositol 3-kinase/protein kinase B/mammalian target of rapamycin (PI3K/AKT/mTOR ) pathways involved in the control of HIF-1 expression in solid tumor tissues. Moreover, a study by Carriere et al. showed that the epithelial-mesenchymal transition of breast cancer cells that are resistant to aromatase inhibitors can be blocked by GI [31]. This effect is partially mediated by the inhibition of zinc finger E-box binding homeobox 1 (ZEB1) expression. 

In summary, particular attention has recently been given to the effects of glyceollins on human health, including their chemo-preventive and antitumoral functions. A growing number of in vitro and in vivo studies have examined these effects [23,24]. In this review, we will first discuss the anticancer properties of glyceollins and their promising effects as new therapeutic agents or dietary supplements in breast and other cancer treatments and then briefly review the potential health benefits of these compounds in other noncancer diseases.

## 2. Synthesis and Structure

**Figure 2:**
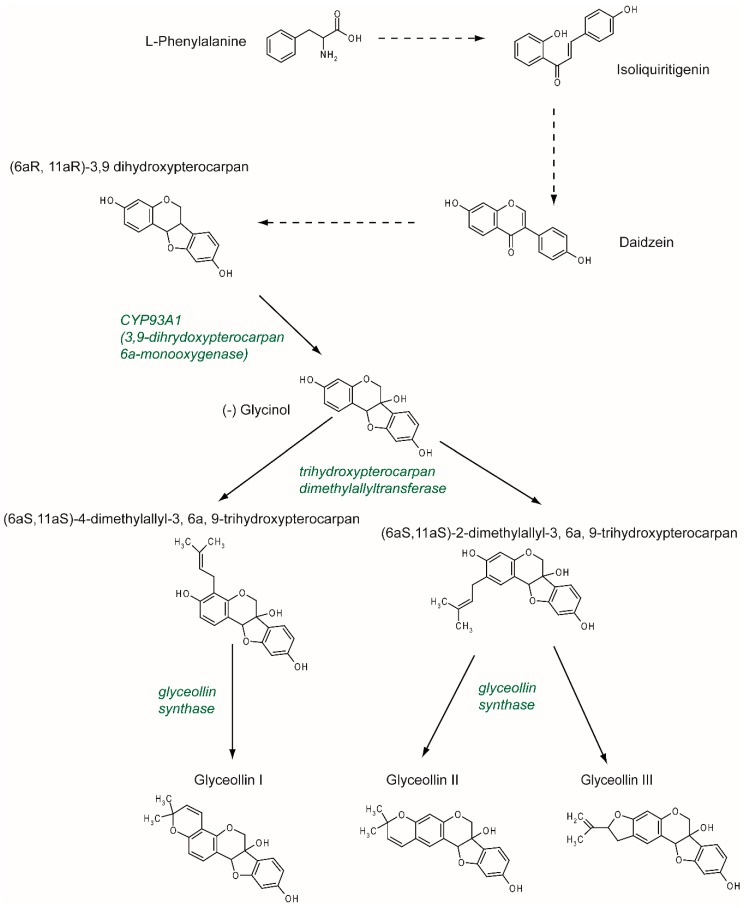
Glyceollin biosynthesis pathway. The isoflavone daidzein serves as the metabolic precursor for the rapid production of glyceollins. Daidzein is produced through the isoflavonoid branch of the phenylpropanoid pathway starting from L-phenylalanine. Under stress, daidzein is transformed into (6aR, 11aR)-3,9 dihydroxypterocarpan. This molecule is metabolized by CYP93A1 into (-) glycinol. Then, a dimethylallyl group is attached to the glycinol at position 2, which leads to the production of glyceollin II and III, or at position 4, which leads to the production of glyceollin I. These two steps are catalyzed by trihydroxypterocarpan dimethylallyl transferase and glyceollin synthase, respectively.

Previously described as hydroxyphaseollin in the early 70s [32,33], glyceollins belong to the family of phytoalexins, which are antimicrobial agents in plants. Glyceollins were characterized in soybeans after inoculation with the pathogen agent *Phytophthora sojae*. There are at least five glyceollins described to date that are produced via a complex metabolic pathway, even though GI, GII, and GIII are the most studied (Figure 2). Glyceollins are produced from the isoflavonoid branch of the phenylpropanoid pathway. Isoflavone daidzein is produced from phenylalanine and stored. Then, in the case of stress, daidzein will be metabolized into glyceollins. After multiple reduction steps, daidzein is transformed into (6aR, 11aR)-3,9 dihydroxypterocarpan. Pterocarpan is characterized by a pyrano-furano-benzene skeleton formed by the coupling of a B ring with the C ring of the isoflavonoid. Then, through the catalytic activity of CYP93A1 (also known as 3, 9 dihydroxypterocarpan 6a monooxygenase), the (6aR, 11aR)-3,9 dihydroxypterocarpan is metabolized into (-)-glycinol [34]. From glycinol, the metabolic pathway of glyceollins differs between glyceollin I and glyceollins II and III. In both cases, the next step consists of connecting a dimethylallyl group to glycinol through the activity of a trihydroxypterocarpan dimethylallyl transferase (also known as a prenyltransferase). For GII and GIII, dimethylallyl is connected to carbon 2, which leads to the production of (6aS, 11aS)-2-dimethylallyl-3, 6a, 9-trihydroxypterocarpan (also known as glyceollidin II) [35]. For GI, the connection of the dimethylallyl group occurs on carbon 4, which leads to the production of (6aS, 11aS)-4-dimethylallyl-3, 6a, 9-trihydroxypterocarpan (also known as glyceollidin I) [36]. In soybeans, approximately 77 prenyltransferases have been identified. Among them, five are induced in response to *Phytophthora sojae,* suggesting that they are involved in phytoalexin biosynthesis. A recent study [37] identified GmPT01 as a prenyltransferase that localized in the plastid and presented root-specific expression. These results suggested that GmPT01 could be involved in GI biosynthesis. The final step of glyceollin biosynthesis consists of the cyclization of the dimethylallyl group by glyceollin synthase. This enzyme belongs to the family of cytochrome P450 and was identified in 1988 [38]. To our knowledge, the specific enzymatic activity that leads to the different glyceollin isomers is not yet known.

Glyceollins are promising molecules in human health, and as such, it is important to decipher the molecular mechanism of the glyceollin biosynthesis pathway. For instance, in a recent study, the co-treatment of soybeans with silver nitrate (AgNO_3_) and with the wall glucan elicitor of *Phytophthora sojae* was shown to specifically enhance the production of glyceollins by not only enhancing the expression of genes encoding specific enzymes, but also by inhibiting the degradation of glyceollins [39].

## 3. Metabolism and Pharmacokinetics

The beneficial antioxidant properties of isoflavones and their derivatives, phytoalexins, have generated a deep interest in their use as nutritional supplements. In terms of bioavailability and pharmacodynamics, the most studied molecule among phytoalexins is resveratrol, which has a high commercial value. However, several recent studies have focused more specifically on the metabolism and pharmacokinetics of glyceollins (Table 1). For instance, in monkeys fed a glyceollin-enriched diet (9 mg/kg/day), the plasma level of glyceollins reached 134.2 nmol/L 4 h after oral administration and was completely undetectable 24 h later [40]. More interestingly, while glyceollins represented approximately 50% of the isoflavones present in the diet used in the previously mentioned study, they did not represent more than 11.6% of the isoflavones found in the plasma 4 h later, due to lower glyceollin absorption or a more rapid elimination [40]. 

The situation seems to be quite different in rodents, where bioavailability is low, but glyceollins appear to remain for a long time. When glyceollins were administered by oral gavage (30 or 90 mg/kg) to rats, glyceollins were found in the plasma starting at 20 min after administration, achieved a peak concentration (118.4 or 159 ng/mL) by 60 min and remained at a stable level until 4 h [41]. Such retention is not common for isoflavones, and a slow clearance may contribute to an accumulation of glyceollins and to a persistent exposure of rodents to glyceollins. However, the difference between the two doses tested (30 or 90 mg/kg) was not found at the plasma level [41]. This suggests that, in rodents, a dose of 30 mg/kg administered orally induces an almost maximal plasma concentration of glyceollins. This information, coupled with the notion of the persistence of glyceollins in rodents, explains the similar effects of different concentrations on the same model. Glyceollins have been shown to improve glucose homeostasis in diabetic rodents, whether orally delivered at a dose of 3 mg/kg [42] or at a dose of 30 mg/kg [41].

Thus, glyceollins have a conventional absorption rate, but their bioavailability is low. A better understanding of the destiny of ingested glyceollins was possible thanks to the identification of conjugated forms of glyceollins by ion scanning using liquid chromatography coupled online with electrospray ionization tandem mass spectrometry (LC-ESI-MS/MS) [43]. This technique thoroughly identifies the conjugated forms of glyceollins. It allowed the identification of glyceollin metabolites derived from the activity of phase I (hydroxylated forms) and phase II (sulfated forms, glutathione or glucuronide conjugates) metabolism in the blood, urine, and feces of orally exposed rats. The three unmetabolized isomers of glyceollins were detected in the feces, suggesting that glyceollins also undergo strong oxidation in the body [43,44]. Thus, first-pass intestinal and hepatic metabolism are barriers to the presence of glyceollins in systemic blood.

The low bioavailability of phytochemicals is notable due to their sulfation and glucuronidation by intestinal enzymes [45,46]. These conjugates, although they facilitate elimination and decrease bioavailability, may also be beneficial for the effects of some molecules. Thus, it has been shown that the sulfated form of resveratrol, which is a biologically inactive, but abundant form after oral intake, is captured by the cells that regenerate active resveratrol [47]. No data exist on such a mechanism with glyceollins.

Glyceollin conjugates have also been described in vitro in CACO-2 cells [48]. In this model, glyceollins (24 h of exposure of up to 100 μM) also decreased the activity of the MRP2 and BCRP transporters (two major apically expressed efflux transporters in the intestine) without significant modification of their expression [49]. This effect can have important consequences on drug efficacy. It can be hypothesized that glyceollins could enhance the absorption of the chemical molecules used in therapy by inhibiting their apical excretion and possibly even enhancing their basolateral efflux, thereby increasing systemic delivery. This effect may reduce prescribed doses of drugs.

Finally, it should be noted that studies have recently been published on the effects of glyceollins on intestinal microbiota. In a mouse model, whose obesity was induced by the diet, the addition of glyceollins to the diet (65 µg/mouse/day for 30 days) modified the microbiota to a less favorable profile for fat absorption, reduced the production of bile, and promoted the formation of short chain fatty acids [50]. All of these effects resulted in improved body composition.

## 4. Anticancer Effects

### 4.1. Estrogen-Dependent Effects

**Figure 3:**
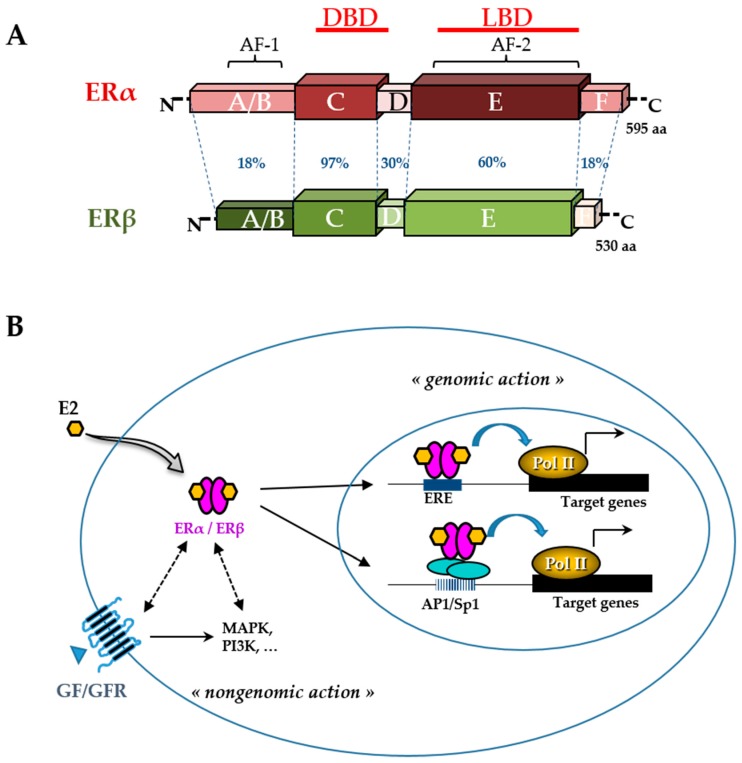
Estrogen receptor (ER) structure and action. The schematic structures of the two human ERα and ERβ and the percentage of homology between the different domains (annotated by the letters A to F) are indicated (**A**). Domains involved in DNA binding (DBD), ligand binding (LBD), ligand-independent transactivation function 1 (AF-1), and ligand-dependent transactivation function 2 (AF-2) are shown. The number of amino acids for each receptor is also indicated on the right side. Estradiol (E2) mediates numerous phenotypic effects in cells by binding to and activating ERs (**B**). E2 enters the cell through the lipid membranes and binds ER, which can be present in the cytoplasm and the nucleus. The activated ER forms a dimer to tightly fix chromatin directly at the estrogen-responsive element (ERE) sites or indirectly at activator protein 1 (AP1) or specificity protein 1 (Sp1) sites. ER is then able to remodel chromatin by recruiting cofactors and activating RNA polymerase II (Pol II), at target genes (genomic action). Besides, ERs can use rapid non-genomic action through the interaction with intracellular kinases (mitogen-activated protein kinase (MAPK), phosphatidylinositol 3-kinase (PI3K),…) and the growth factor (GF) receptor (GFR) pathways.

Like other phytoestrogens, glyceollins have a similar structure to that of the natural hormone, 17β-estradiol (E2). Therefore, their effects in estrogen-dependent diseases have been the most investigated. Breast cancer is the most frequent type of cancer in women worldwide, with nearly 1.7 million new cases diagnosed in 2012 [51]. Among the different types of breast cancer, the most common is estrogen receptor (ER)-positive cancer, which represents approximately 75% of the diagnosed cases of breast cancer [52]. The ER belongs to the nuclear receptor superfamily and is divided into two subtypes, ERα and ERβ (Figure 3). These receptors, which function primarily as ligand-activated transcription factors, regulate various cellular functions, such as proliferation, survival, differentiation, and apoptosis [53]. They are characterized by distinct domains comprising a conserved zinc finger DNA binding domain (DBD), a ligand binding domain (LBD), and two transactivation functions (AF). ERs typically alter the transcription of target genes by direct interaction with an estrogen-responsive element (ERE) and by recruiting transcription factors involved in chromatin remodeling [54]. However, many E2 target genes are devoid of an ERE in their promoter. In this case, ERs can modulate their transcription by establishing protein interactions with activator protein 1 (AP1) or specificity protein 1 (Sp1) transcription factors [55]. In addition, there are multiple levels of cross-talk between ERs and other intracellular pathways. Indeed, although ERs are mainly in the nucleus, a small amount of ERs can be present in the cytosol or near the plasma membrane. They are capable of interacting and activating various signal transduction cascades, such as mitogen-activated protein kinase (MAPK), protein kinase C, and phosphatidylinositol 3-kinase (PI3K). This type of interaction could explain the so-called ”non-genomic actions” of estrogen [53] (Figure 3). ERα, the major isoform in breast tissue, plays an essential role in normal mammary gland development and function as well as in breast cancer initiation and growth [56]. ERα is bound by the natural hormone, E2, which has a pleiotropic effect and is responsible for the proliferation and survival of breast epithelial cells. Therefore, in the case of ER-positive breast cancer, ERα is a good prognostic marker and a prime target for therapy. The antagonist effect of glyceollins on ER activity was first discovered in 2001 in both HEK 293 and MCF-7 cells [27]. More recent studies confirmed this antiproliferative effect of glyceollins in E2-dependent proliferative cell models [25,28]. Not only were their molecular modes of action better described, but also the glyceollin isomers were studied in isolation.

Some phytoestrogens, such as coumestrol, daidzein, and genistein, are partial agonists of ERs. They have ER-agonist activity in low-estrogen conditions, but ER-antagonist activity in high-estrogen conditions. Unlike some phytoestrogens, the mixture of glyceollins shows only antagonist effects on ERs in low-estrogen conditions [27,28]. These compounds have a greater affinity for ERα than ERβ [27], which shows their potential to treat breast cancer, where ERα is the major isoform. 

GI is the most potent antiestrogenic isomer in the glyceollin mixture [28,57]. GI has the strongest capacity to bind to ERα due to its highest binding affinity for ERα. Examinations of the interaction between GI, GII, and GIII and the ERα ligand binding cavity in docking studies demonstrated that GI interacts with ERα in a way that is similar to tamoxifen, whereas GII and GIII can only bind to ERα in a completely different way [28]. Among glyceollins, GI is also the strongest inhibitor of ER transcriptional activity and colony formation in MCF-7 and BG-1 cells [28]. Its effects are different from that of its estrogenic precursor, glycinol [58]. Among GI enantiomers, (+)-GI (synthetic enantiomer) slightly increased ERE activity, while (-)-GI (natural enantiomer) decreased the activity of both ER subtypes stimulated by E2, demonstrating potent anti-estrogenic properties [57]. 

**Figure 4:**
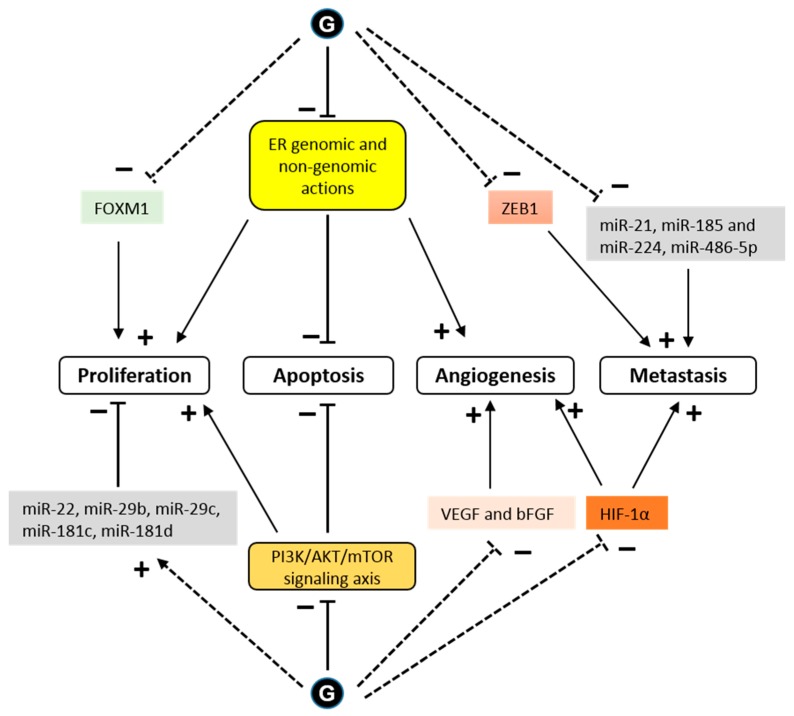
Signaling pathways modulated by glyceollins in the context of cancer cells. Glyceollins (G) have been shown to directly interact with the estrogen receptor (ER), exerting antagonistic effects on ER-dependent pathways. This anti-estrogenic effect of glyceollins prevents E2-dependent proliferation and angiogenesis. In addition, glyceollins induce cell apoptosis by direct ER genomic or nongenomic (membrane-initiated) effects. The expression of forkhead box M1 (FOXM1), a key regulator of the cell cycle, is downregulated by glyceollins. Cell growth and apoptosis can also be affected by glyceollins through ER-independent pathways. Glyceollins inhibit the activity of cytoplasmic kinases, such as the phosphatidylinositol 3-kinase/protein kinase B/mammalian target of rapamycin (PI3K/AKT/mTOR) signaling axis. Glyceollins repress the expression of growth factors, such as vascular endothelial growth factor (VEGF) and basic fibroblast growth factor (bFGF), and promote the expression of microRNAs (miRs) that act as tumor suppressors. Glyceollins also inhibit cell invasion and metastasis. These effects could be partially mediated by the inhibition of zinc finger E-box binding homeobox 1 (ZEB1) and hypoxia inducible factor 1 (HIF-1) expression as well as of microRNAs that enhance tumorigenesis (see text for references). Solid and dashed lines indicate direct and indirect effects, respectively. (+) indicates promoting effect and (-) indicates inhibiting effect.

As a transcription factor, ERα either directly or indirectly regulates the expression of many genes that are involved in cell growth and proliferation. Glyceollins inhibited the expression of the E2 target genes, PgR and CXCL12 (SDF-1), in MCF-7 cells [25,28]. A transcriptomic assay determined that the antiproliferative effect of glyceollins in ER-positive breast cancer cells was achieved through the ER and forkhead box M1 (FOXM1) factor pathway [25]. Glyceollins downregulated FOXM1, which is a well-known key regulator of the cell cycle, involved in G1/S and G2/M transitions (Figure 4).

Glyceollins can suppress ER transcriptional activity independently of the ligand binding domain and ERα phosphorylation. Glyceollins suppress the phosphorylation of proteins known to crosstalk with ER signaling, specifically protein S6 kinase, 70 kDa (p70S6K) [59]. As one of the best characterized downstream targets of mTOR, p70S6K is an important regulator of cell size, protein translation, and cell proliferation [60].

The antiestrogenic effect of glyceollins was also demonstrated in vivo. Indeed, mice xenografted with MCF-7 breast cancer cells and injected with a mixture of glyceollins showed a decrease in tumor growth [26,28]. Synthetic GI and GII decreased E2-dependent proliferation of mammary glands in ovariectomized mice [25]. In female monkeys, a glyceollin-enriched diet inhibited breast proliferation stimulated by E2 [40]. The expression of two ER-target genes in the breast epithelium, TFF1 and PgR, was markedly lower in the group with a glyceollin diet compared to that in the group treated with E2 alone.

The effect of glyceollins in other E2-dependent cell types or tissues was also investigated. GI inhibited colony formation of ovarian cancer BG-1 cells [28]. With their antiestrogenic action, glyceollins can inhibit the growth of human ovarian cancer xenografts [26]. After 20 days of the experiment, a treatment with a glyceollin mixture combined with E2 showed a reduction in BG-1 ovarian tumor volume (73.1%) when compared to E2 alone. These tumor-inhibiting effects corresponded to significantly lower E2-induced PgR expression in the tumors. While the use of tamoxifen or certain phytoestrogens in postmenopausal women with breast cancer is a concern because of the side effects of their estrogenic action on other tissues, such as the uterus [61], glyceollins did not show any estrogenic effects on the uterine morphology and partially antagonized the uterotropic effects of estrogen in nude mice after 20 days of treatment [26]. However, in our recent study, a brief treatment via the injection of GI and GII alone for 3 days showed that these compounds had no antagonistic effect on the trophic action of E2 in mouse uteri [25]. The ability of glyceollins to function as an estrogen antagonist in the uteri of mice after a long-term treatment is a distinct advantage when compared with that of other compounds.

Furthermore, glyceollins exerted growth inhibitory effects on the human androgen-responsive prostate cancer cell line, LNCaP, by leading to G1/S arrest. Interestingly, this effect of glyceollins appeared to be mediated through the modulation of an estrogen-, but not androgen-mediated pathway [62].

### 4.2. Estrogen-Independent Effect

In addition to their ER-dependent antitumor effect, glyceollins could also suppress mammary tumorigenesis through ER-independent pathways. For instance, in vitro, glyceollins decreased the proliferation of the ER-negative breast cell line, MCF10A [25], and in vivo decreased MDA-MB-231 and MDA-MB-468 breast tumor volume in xenografted mice [63]. 

One potential mechanism for metastatic spread is the epithelial to mesenchymal transition (EMT) [64]. Characteristic of these EMT cells is a loss of E-cadherin expression and high expression of the transcription factor, ZEB1 [65]. The ZEB1 transcription factor, known as an inducer of EMT in cancer metastasis, acts through the transcriptional repression of E-cadherin. When letrozole-resistant breast cancer cells (LTLT-Ca) were treated with GI, they exhibited morphological characteristics similar to an epithelial phenotype, and the GI treatment decreased proliferation by increasing E-cadherin and decreasing ZEB1 [31]. These results demonstrated that GI could reverse EMT. In another study in MDA-MB-231 cells, glyceollins significantly increased miRNAs involved in EMT (miR-22, miR-29b, miR-29c, miR-30d, miR-34a, and miR-195) and tumor suppressors (miR-181c and miR-181d). There was also a significant decrease in the expression of oncomiRs promoting tumorigenesis (miR-21 and miR-193a-5p), oncomiRs promoting metastasis, such as miR-185, miR-224, and miR-486-5p, which are involved in cell migration and invasion, and miR-542-5p, which is involved in the maintenance of a mesenchymal phenotype (Figure 4).

The formation of microvessels is a critical step in the progression of cancer [66]. Multiple growth factors and cytokines existing in the tumor microenvironment contribute to angiogenic processes. Among them, VEGF and basic fibroblast growth factor (bFGF) are the major angiogenic factors induced by hypoxia. In vitro, glyceollins inhibited both VEGF- and bFGF-induced angiogenesis [29]. In vivo, glyceollins strongly blocked angiogenesis in zebrafish and chick embryos as well as in mice xenografted with lung cancer cells [29]. This effect of glyceollins could be useful not only in the case of cancers, but also for other angiogenesis diseases. 

HIF-1α is a transcription factor that is constitutively expressed. Under normoxic conditions, HIF-1α is rapidly degraded by the proteins, prolyl hydroxylase domain (PHD) and factor inhibiting HIF (FIH-1) [67]. Under hypoxic conditions, HIF-1α is stabilized and exerts its transcriptional activity. The presence of active heat shock protein 90 (HSP90) is also necessary for the rapid accumulation and activation of HIF-1α in both normoxic and hypoxic conditions [68]. There is an association between the stability of HIF-1α and tumor growth [69]. Lee et al. showed that glyceollins potently inhibited HIF-1α synthesis and decreased its stability by blocking the PI3K/AKT/mTOR pathway and HSP90 binding activity, respectively, under hypoxic conditions [30]. However, surprisingly, our recent study showed that glyceollins induced HIF-1α under normoxic conditions [25]. HIF-1α is controlled by numerous stimuli, including reactive oxygen species (ROS) [70], whose production is activated by glyceollins at high concentrations [71]. The effect of glyceollins on HIF-1α could be due to its role in increasing the expression of REDD1, an mTORC1 inhibitor [25]. The inhibition of mTORC1 could be a factor involved in the antiproliferative effects of glyceollins through altering the PI3K/AKT/mTOR pathway (Figure 4).

The anticancer effect of glyceollins may be linked to their antioxidant effect, as discussed hereafter. Pretreatment or cotreatment with glyceollins protected mice from 7,12-dimethylbenz(a)anthracene-induced mammary tumorigenesis by reducing tumor formation and increasing the survival rate [72]. This protective effect was mainly associated with their potential to induce phase 2/antioxidant enzymes that play an essential role in enhancing the hydrophilicity of exogenous carcinogens to excrete them into bile or urine. However, at high concentration, glyceollins stimulated the production of ROS, which are possibly responsible for the apoptotic activity of the compounds [71]. Treatment with glyceollins at a high dose decreased cell viability and the mitochondrial membrane potential and increased DNA fragmentation. With their apoptotic potential, glyceollins could be exploited as antitumorigenic agents.

## 5. Other Effects in Noncancer Diseases

### 5.1. Osteoporosis

Osteoporosis is the most common bone disease in humans, representing a major public health problem, especially in menopause aged women [73]. Osteoporosis is defined pathologically by an imbalance between bone resorption and bone regeneration [74]. Therapeutic compounds for osteoporosis are divided into two main groups: Anti-resorptive drugs and anabolic drugs [74,75]. Anti-resorptive drugs reduce bone loss while anabolic drugs increase bone formation. E2 reduces osteoporosis through both anti-resorptive and anabolic mechanisms, but its use is disputed due to concerns about the risk of developing breast and endometrial cancers after this therapy [76,77]. Phytoestrogens can lead to promising alternative therapeutic strategies because they have shown the capacity to inhibit the bone resorption activity of osteoclasts, to stimulate osteogenic differentiation, and to increase the maturation of bone marrow-derived mesenchymal stem cells and osteoblasts [78]. A recent study from Bateman et al. examined the effect of glyceollins on the osteogenesis of adipose-derived stromal cells and bone marrow stromal cells [76] (Table 2). The treatment of these cell models with GI and GII showed increased calcium deposition relative to that of vehicle–treated cells. Interestingly, GII was more efficient in osteogenesis than E2 and GI. This effect of GII was partially inhibited by the antiestrogen, fulvestrant, indicating that the osteoinductive effect of GII is partly mediated by ER pathways. An analysis of gene expression demonstrated that GII increased the expression of genes involved in osteogenic differentiation in a similar manner to E2. Thereby, glyceollins are promising for development to become an anabolic drug in osteoporosis treatment. 

### 5.2. Glucose and Lipid Metabolism

Glyceollins have shown potential benefits in glucose and lipid metabolism (Table 2). In mice, the intake of a diet with 0.41 mg/day of glyceollins for 8 weeks decreased fasting serum glucose, increased fasting serum insulin, and decreased serum nonesterified fatty acids in streptozotocin-induced diabetic mice [42]. These effects of a decrease in blood glucose were also observed in prediabetic rats [41]. A diet with glyceollins reduced insulin resistance by as much as did rosiglitazone, a commercial insulin sensitizer [42]. In more detail, in the liver, the glyceollin diet improved insulin signaling and glucose metabolism by increasing the phosphorylation of protein kinase B (Akt) and AMP-kinase and by decreasing the phosphorylation of acetyl-CoA carboxylase and the expression of phosphoenolpyruvate carboxykinase [42]. In adipocytes, glyceollins improved insulin-stimulated glucose uptake and decreased triacylglycerol accumulation [79]. The stimulation of glucose uptake was due to an increase in glucose transporter 4 (GLUT4) transporters [41]. In the pancreas, the glyceollin diet partly increased the insulin secretion capacity by increasing the β-cell area and mass in islets of Langerhans [42]. In insulinoma cells, glyceollins increased the insulin level and alleviated the palmitate-induced impairment of β cell function and apoptosis via attenuation of endoplasmic reticulum stress [79]. Glyceollins decreased the expression of genes involved in fatty acid synthesis that are normally decreased by estrogen.

In the golden Syrian hamster, which have similar lipid profiles and a similar level of susceptibility to dietary cholesterol as humans, glyceollin supplementation significantly decreased plasma very low-density lipoprotein (VLDL), hepatic cholesterol esters, and the total lipid content. Moreover, the glyceollin diet also altered the expression of genes related to cholesterol metabolism in the liver [80].

In female surgically postmenopausal cynomolgus macaques, diet containing glyceollins for 3 weeks resulted in lower serum total cholesterol level, specifically low-density lipoprotein (LDL) and VLDL, and increased serum triglycerides level [81]. For gene expression in mammary biopsies, the glyceollin diet affected genes involved in lipid and carbohydrate metabolism, notably it upregulated peroxisome proliferator-activated receptor (PPAR)-γ, adiponectin, leptin, lipin 1, and lipoprotein lipase [81].

Together, these results supported that glyceollins have beneficial effects in glucose and lipid metabolism. A diet containing glyceollins may help to improve metabolic diseases. 

### 5.3. Antioxidant Effect

The oxidation of nutrients for a biological energy supply is essential to life for all aerobic organisms [95]. However, cells that benefit from oxidative respiration are also burdened by ROS [95]. However, healthy cells balance the formation and elimination of ROS [95]. When the concentration of free radicals exceeds a critical level and the ROS-homeostasis is disturbed, oxidative stress occurs [95]. Oxidative stress plays a critical role in many diseases, such as diabetes, cardiovascular and neurodegenerative diseases, chronic inflammatory diseases, and cancer [96]. Several compounds from natural sources, such as quinoa and amaranth grains [97], nut [98], cranberry [99], and soybeans [100], benefit health through their antioxidants activities. As previously discussed in Section 4.2 about the antitumor estrogen-independent effects, the antioxidant activity of glyceollins has been demonstrated in several studies (Table 2). Glyceollins showed strong reducing power, inhibited lipid peroxidation via their large capacity to scavenge radicals, and suppressed H_2_O_2_-induced ROS production in hepa1c1c7 cells [82]. Moreover, glyceollins have been reported to induce phase 2 detoxifying enzymes [83,84]. Experimental and docking analyses suggested that this action occurred through promotion of the nuclear translocation of the nuclear factor (erythroid-derived 2)-like 2 (Nrf2) by disrupting Keap1-Nrf2 complex formation [83,84]. In mice, glyceollin treatment increased NAD(P)H:quinone oxidoreductase 1 (NQO1) activity in the kidney, liver, and large intestine [72]. In the rat, glyceollins inhibited lipid peroxidation in the liver, kidney, and brain tissue extracts [82]. Since oxidative stress can be considered as one of the causes of many common human pathologies and of the aging process [96], glyceollins, with their antioxidant effects in vitro and in vivo, could be further studied to become a preventive therapy for oxidative stress-caused diseases. 

### 5.4. Effect on the Central Nervous System

Several studies showed the neuroprotective effects of glyceollins on the central nervous system (CNS) (Table 2). Recently, Seo et al. examined the neuroprotective effect of glyceollins through the activation of Nrf2 in two neuronal cell models [85]. The transcription factor, Nrf2, is the major regulator of the expression of different antioxidant enzymes, such as heme oxygenase 1 (HO-1). Using mouse primary cortical neurons treated with glutamate, they showed that glyceollins reduced glutamate-induced cytotoxicity in neurons expressing wild-type Nrf2, but not in Nrf2 knockout neurons. Likewise, glyceollins reduced the production of ROS, which are involved in neurodegeneration, by stimulating the expression of Nrf2 and HO-1 in a hippocampal neuronal HT22 cell line. In this model, glyceollins significantly reduced ROS production after the cells were exposed to glutamate. Moreover, in a mouse model of amnesia, glyceollins improved mnemonic and cognitive deficits caused by scopolamine in wild-type, but not in Nrf2 knockout mice. These results suggest that glyceollins’ actions are likely mediated by the stimulation of the Nrf2 / HO-1 signaling pathway [85].

Estrogens regulate numerous functions in the brain, including reproduction, food intake, cognition, neuronal synaptic plasticity, and pain perception [87]. As phytoestrogens, glyceollins may have effects in brain functions through ERs. Bamji et al. investigated the effect of glyceollins in a female mouse brain using microarray experiment [86] and paired-end RNA sequencing (RNA-seq) [87]. They showed that the actions of glyceollins in the CNS could be mediated by ER-dependent and ER-independent mechanisms depending upon the target gene. The transcripts regulated by E2 and glyceollins alone or in combination annotated to similar pathway maps and network in both microarray and RNA-seq experiments. In microarray, they showed that glyceollins upregulated genes involved in neurogenesis, synaptic plasticity, and tissue development [86]. In particular, the glyceollins downregulated the expression of the gene encoding peptidylprolyl isomerase A (PPIA), which regulates several biological processes, including inflammation and apoptosis. PPIA stimulates neurodegeneration and the loss of synaptic connections by activating NF-κB and metalloproteinase 9 [101]. In the RNA-seq experiment, glyceollins upregulated transcripts involved in signal transduction pathways, regulation of nerve impulse, cytoskeletal remodeling, and hormone signaling, and downregulated transcripts involved in developmental neurogenesis, synaptogenesis, and cell adhesion [87]. Although these results suggested that glyceollins may potentially have neuroprotective effects by regulating the gene expression related to these pathways, the mechanism through which glyceollins affects gene expression in the brain is still unclear.

**Figure 5:**
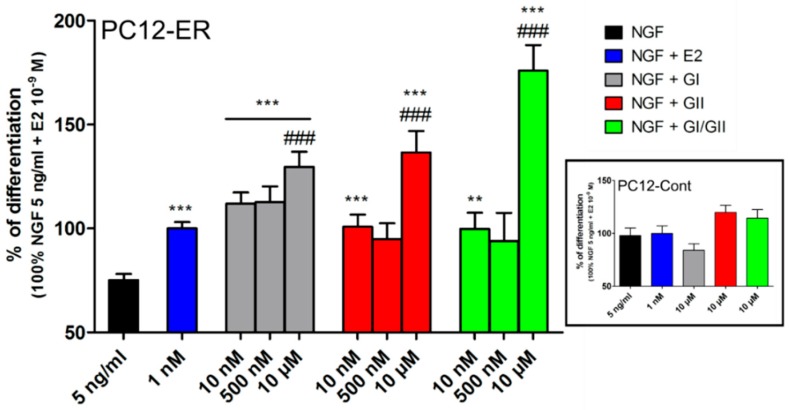
Glyceollins enhance the NGF effect on PC12-ER differentiation. PC12-ER transfected with ERα and PC12-Cont transfected with empty plasmid (cartridge) cells were treated for 72 h with 5 ng of nerve growth factor (NGF) associated with solvent (black), E2 10^-9^ M (blue), various concentrations of glyceollin I (GI, gray) or glyceollin II (GII, red), or a mixture (1:1) of both GI and GII (green). PC12 cells were then photographed to determine the percentage of cells bearing neurites. The results are expressed as the percentage of cells bearing neurites in the presence of NGF + E2. The results are the mean of at least three independent experiments ± SEM. ** *p*-value < 0.01 and *** *p*-value < 0.001, with one-way ANOVA and Bonferroni’s post hoc test used to compare the control cells (NGF + solvent) with the other treatments. ### *p*-value < 0.001, with one-way ANOVA and Bonferroni’s post hoc test used to compare the NGF + E2-treated cells with the other treatments.

Using an in vitro cell model for neuronal differentiation [102], we tested the ability of glyceollins to enhance nerve growth factor (NGF)-induced neuritogenesis in the PC12 cell line (Figure 5). We have previously shown that PC12 cells stably transfected with ERα (PC12-ER) are able to develop a pseudoneuronal phenotype (neurite outgrowth) when treated with NGF [103,104,105]. As shown in Figure 5, our data clearly showed that GI and GII fully contributed to the neuritogenesis effect of NGF in PC12-ER (Figure 5), but not in PC12 control cells (PC12-Cont) that do not express ERα (Figure 5 –cartridge). This indicates that the effect of glyceollins depends on the presence of ER. It is interesting to note that Zimmermann et al. [28] previously reported that glyceollins strongly increased the expression of the nerve growth factor receptor (NGFR) gene, a member of the tumor necrosis factor receptor superfamily. This may explain the effect of glyceollins on neurite outgrowth in the PC12 model. Thus, although many more studies are needed to understand the effects of glyceollins on the CNS, these compounds might be used in the development of therapeutic treatments for CNS diseases.

### 5.5. Anti-Inflammatory Activity and Cardiovascular Disease

Inflammation is a complex biological protective response against bodily injury [106]. However, continuous and chronic inflammation is linked to many chronic diseases, including cancer, cardiovascular diseases, obesity, and type 2 diabetes [107,108]. Glyceollins decreased inflammatory reactions caused by LPS in a murine macrophage cell line (RAW264.7) by inhibiting nitric oxide (NO) production and inflammatory mediators release [88,89]. Glyceollins inhibited IL-6 release and reduced the expression of iNOS and COX2 [88]. Moreover, glyceollins inhibited the expression of other pro-inflammatory cytokines, such as IL-1β, IL-18, and TNF-α, and upregulated the expression of anti-inflammatory cytokines, including IL-10, in the RAW264.7 cell line [89]. This action was found to occur through the NF-κB pathway. In vivo, glyceollins reduced mouse ear swelling caused by TPA [88]. 

Moreover, glyceollins have anti-septic effects via inhibition of the high mobility group box 1 (HMGB1) signaling pathway [90]. The biological activity of HMGB1 depends on its location, context, and post-translational modifications. However, a high level of circulating HMGB1 has been found in various diseases, particularly severe inflammatory diseases [109]. In the human umbilical vein endothelial cell line, glyceollins reduced HMGB1 protein levels induced by LPS. Consequently, glyceollins decreased the HMGB1-mediated pro-inflammatory and inflammatory response as well as vascular barrier disruption [90]. In mice, glyceollin treatment reduced serum HMGB1 level, HMGB1-induced vascular barrier disruption, and mortality [90]. Hence, glyceollins could be potential therapeutic agents for severe vascular inflammatory diseases, such as sepsis and septic shock. 

The inflammatory process is often implied in cardiovascular diseases [107]. Glyceollins have shown their beneficial effects in these diseases not only through their anti-inflammatory effect, but also through other pathways (Table 2). GI reduced vascular contraction in an endothelium-independent manner partly through the inhibition of the RhoA/Rho-kinase signaling pathway in rat aortic rings [92]. Since the RhoA/Rho-kinase pathway plays a crucial role in cardiovascular diseases, such as vasospasm, arteriosclerosis, ischemia/reperfusion injury, hypertension, and heart failure [110], GI, with its vasorelaxant activity, can be studied as a cardiovascular therapeutic agent. 

Platelet-derived growth factor (PDGF)-BB can induce the abnormal proliferation and migration of aortic smooth muscle cells (ASMC), which play an important role in the formation and development of atherosclerosis [111]. Kim et al. showed that glyceollins could inhibit the proliferation and migration of human ASMC induced by PDGF-BB [91]. Glyceollin treatment diminished the increased cell number and DNA synthesis caused by PDGF. This effect came from the capacity of glyceollins to block the PDGF-induced G_0_/G_1_ to S phase transition of the cell cycle, downregulating the expression of cyclin-dependent kinase (CDK)2 and cyclin D, and upregulating the expression of CDK inhibitors, such as p27^kip1^ and p53. Moreover, glyceollins inhibited the PDGF-induced dissociation of actin filaments and cell migration.

Furthermore, the antioxidant and cholesterol-lowering effects of glyceollins can also have an effect in cardiovascular diseases.

Briefly, glyceollins have anti-inflammatory activities through many different pathways. Therefore, they could be potential therapeutic agents for several inflammatory diseases, such as cardiovascular diseases, obesity, and type 2 diabetes. 

### 5.6. Anti-Melanogenesis Activity

Melanin constitutes a group of natural dark pigments produced by a group of specialized cells known as melanocytes. In human skin, melanin plays an essential role in protecting the skin from DNA damage caused by ultraviolet radiation [112]. However, the abnormal accumulation of melanin causes dermatological problems, such as freckles, solar lentigo, melisma, postinflammatory melanoderma, and cancer [113]. In B16 melanoma cells, glyceollins decreased melanin synthesis by inhibiting melanogenic enzymes, such as tyrokinase and tyrosinase-related protein (TRP)-1 [93]. Additionally, glyceollins diminished intracellular cyclic AMP (cAMP) levels stimulated by α-melanocyte-stimulating hormone [93]. Melanogenesis is regulated by numerous factors, among which the stem cell factor (SCF) and the microphthalmia-associated transcription factor (MITF) play an important role. SCF plays a critical role in regulating the life cycle of human melanocytes, and MITF controls the expression of the melanogenic enzymes, tyrokinase, TRP-1, and TRP-2 [114]. In B16F10 melanoma cells, glyceollins attenuated melanin synthesis, tyrosinase activity, and c-kit and Akt phosphorylation induced by SCF [94]. Moreover, glyceollins significantly decreased the expression and activity of MITF and cAMP induced by SCF [94]. 

The anti-melanogenesis activity of glyceollins was also confirmed in zebrafish embryos [94]. Glyceollin treatment decreased the pigmentation of the embryos, melanin synthesis, and tyrosinase activity of the embryos. This treatment also dramatically decreased the expression of *Sox10*, a pigment cell-specific gene, in the neural tubes of the trunk region of the embryos. These results revealed that glyceollins could be useful for the treatment of hyperpigmentation problems or skin-whitening agents for cosmetic use. 

## 6. Conclusions

Epidemiological studies have emphasized that plant-based diets (vegetables, fruits, herbs, and whole grains) may be beneficial for the prevention of certain chronic diseases and may moderately decrease the risk of developing certain cancers [4,9,115]. Because of their various structures, polyphenols are involved in a large array of biological functions, including antioxidant, anti-inflammatory, antiproliferative, and antiangiogenesis effects [1]. Consequently, these properties are attracting increasing interest for the prevention and treatment of cancer and inflammatory, cardiovascular, and neurodegenerative diseases [116]. 

In this review, we summarized diverse aspects of glyceollins and their molecular actions, indicating that these natural compounds can be beneficial to human health by reducing the incidence of numerous diseases. At the molecular level, glyceollins have been shown to interact with ERs and act as weak estrogenic/anti-estrogenic compounds in a cell-dependent manner. It was suggested that glyceollins can behave like selective ER modulators (SERMs) that have partial agonist and antagonist properties depending on the ER isoform or cellular context [117]. In our previous study [25], glyceollins acted essentially as antiestrogenic compounds via the direct inhibition of ER activities in the context of breast cells. Thus, the reason for this conflicting finding could be explained by the use of different cell models in these two studies [25,117]. Nevertheless, consistent with the possible SERM activity of these compounds, we showed here that GI and GII enhanced the differentiation of PC12 cells, as E2 did. Our genome-wide microarray performed with the MCF-7 breast cell line treated with synthetic GI and GII, alone or together with E2, identified the FOXM1/ERα and HIF-1α/HIF-2α signaling axes as the two major pathways affected by glyceollins [25]. This result could explain the antiproliferative effects of glyceollins on ER-positive and ER-negative cell lines. To date, two studies have used xenograft mouse models that were injected with breast or ovary carcinoma cells and treated with E2 and a mixture of glyceollins. The results showed that glyceollins inhibited E2-stimulation of tumor growth, suggesting that they might be helpful in breast and ovarian carcinoma therapies [26,28]. Furthermore, in mice and zebrafish models, glyceollins caused a reduction in microvessel density, suggesting a potential preventive effect for hypervascularization caused cancers [29]. Further studies on the antiproliferative effects of glyceollins in hormone-dependent and -independent cancers using nude mice xenografted with tumor cells and treated orally with glyceollins in combination with traditional therapeutic agents (e.g., Tamoxifen, Fulvestrant, Everolimus) would be beneficial. This characterization would be a first step in determining whether the combined action of glyceollins and traditional therapeutic agents is effective and whether it is possible to decrease the dose of chemotherapy. Furthermore, a precise assessment of the mechanisms by which these components prevent cancer cell growth as well as their potential actions on the epigenetic programming of ER-target genes should be investigated. In addition to their anticancer properties in prostate, breast, and ovarian carcinomas [26,62], glyceollins have also shown antioxidant and anti-inflammatory actions as well as inhibitory effects on tyrosine kinase activities, Akt phosphorylation, and ribosomal protein S6 kinase (p70S6K) phosphorylation [59]. Glyceollins could impact the metabolism of drugs and xenobiotics by regulating the expression of genes encoding cytochrome P450 phase I and other detoxifying enzymes ([25] and unpublished data). They have neuroprotective effects in vivo and in neuronal cell models. Additionally, as we have reported here, glyceollins enhance neuronal differentiation, resulting in neurite outgrowth in PC12 cells. This action depends on the presence of ER.

Although much work has been done, much work also remains to be done before we can consider using these compounds as preventive or therapeutic agents in human pathologies. Indeed, little is known about the bioavailability and pharmacokinetics of glyceollins in different tissues. Some polyphenols, such as resveratrol or apigenin, are poorly bioavailable and are rapidly metabolized in the intestine or liver [118,119]. More data are also needed in toxicological studies to determine the effective and nontoxic doses in vivo. In addition, to date, no clinical or epidemiological studies have been done about glyceollins treatment. So, clinical trials on the use of glyceollins as supplements are required to obtain accurate data on the efficacy of these compounds and on the correct dosage for biomedical applications in human health.

## Figures and Tables

**Table 1:** Bioavailability of glyceollins.

Animal Model	Treatment	Sample	Time	Method of Measure	Major Results	References
Female monkey (Macaca fascicularis)	Diet containing glyceollin mixture ^1^, 134 mg/day representing 50% of total isoflavonoids in the diet	Plasma	4 h and 24 h post administration (postad)	Liquid chromatographic-photodiode array mass spectrometric analysis	Plasma concentration of glyceollins: - 4 h: 134.2 ± 34.6 nmol/L, representing only 11.6 % of the plasma isoflavonoids level - 24 h: Undetectable ˂ 1 nmol/L	Wood et al. [40]
Male ZDSD/Pco rat	Glyceollin mixture, gavage, 30 and 90 mg/kg	Plasma	20, 60, 120 and 240 minutes (min) postad	HPLC-electrospray ionization-MS/MS	Plama concentration of glyceollins: - 20 min: Starts to be detectable - the next 220 min: Remains stable For dose 30 mg/kg: Ranges from 81.2 to 118.4 ng/mL For dose 60 mg/kg: Ranges from 118.2 to 159.0 ng/mL - 60 min: Peak concentration	Boué et al. 2012 [41]
	Glyceollin mixture, gavage, 90 mg/kg/days for 2 weeks	Plasma, feces, and urine	Plasma: 3 h postad Feces: Once daily for 2 weeks Urine: 24 h collection postad a single dose	Precursor and product ion scanning using liquid chromatography coupled online with Electrospray ionization tandem mass spectrometry	- Rapidly absorption, glyceollins undergo phase I and phase II metabolism in the small intestine and the liver - Metabolites of glyceollins were identified in the plasma, the urine, and the feces Phase I conjugation: Epoxidation, hydroxylation… Phase II conjugation: Sulfate and glucuronide conjugations…	Quadri et al. 2013 [43], Quadri et al. 2014 [44]

^1^ Glyceollin mixture contains glyceollin I, glyceollin II and glyceollin III.

**Table 2:** Glyceollins effects in noncancer diseases.

Diseases or Functions	Cell Line/Animal Model	Treatment	Major Effects	References
Osteoporosis	Adipose-derived stromal cells and bone marrow stromal cell line	GI ^1^ and GII ^2^	- GI and GII: Increase calcium deposition - GII: Stronger than E2 and GI, increase the expression of genes involved in osteogenic differentiation in a similar manner to E2	Bateman et al. 2017 [76]
Glucose and lipid metabolism	Enteroendocrine NCI-H716 cell line	Glyceollin mixture ^3^	- Enhance GLP-1 ^4^ secretion to increase insulinotropic actions	Park et al. 2010 [79]
	3T3-L1 adipocyte cell line	Glyceollin mixture	- Increase both insulin-stimulated and basal glucose uptake - Increase glucose transporter GLUT4 ^5^ level - Decrease triacylglycerol accumulation	Boué et al. 2012 [41], Park et al. 2010 [79]
	Insulima Min6 cell line	Glyceollin mixture	- Decrease apoptosis - Enhance insulinotropic actions	Park et al. 2010 [79]
	Diabetic male C57BL6J mouse	Fermented soybeans containing glyceollins	- Decrease blood glucose level - Increase hepatic glycogen accumulation - Decrease triglyceride storage	Park et al. 2012 [42]
	Prediabetic male ZDSD/Pco rat	Glyceollin mixture	- Decrease blood glucose level	Boué et al. 2012 [41]
	Male golden Syrian hamster	Diet containing glyceollins	- Reduce plasma VLDL ^6^, hepatic cholesterol esters, total lipid content - Alter expression of the genes related to cholesterol in liver	Huang et al. 2013 [80]
	Female monkey (Macaca fascicularis)	Diet containing glyceollins	- Decrease serum total cholesterol, specifically LDL ^7^ and VLDL, increase serum triglycerides - Upregulate genes expression of PPAR ^8^-γ, adiponectin, leptin, liptin 1, lipoprotein lipase and triglyceride	Wood et al. 2012 [81]
Oxidative stress	Tests in vitro	Glyceollin mixture	- Have ferric-reducing antioxidant power, radical scavenging activities	Kim et al. 2010 [82]
	Hepa1c1c7/ BPRc1 cell line	Glyceollin mixture	- Inhibit H_2_O_2_-induced ROS ^9^ production - Induce Nrf2-mediated phase 2 detoxifying enzymes - Activate Nrf2-signaling pathway under oxidative stress	Kim et al. 2010 [82], Kim et al. 2011 [83] Jung et al. 2013 [84]
	Female C57BL/6J mouse	Glyceollin mixture	- Increase NAD(P)H:quinone oxidoreductase 1activity in kidney, liver, and large intestine	Kim et al. 2015 [72]
	Rat	Glyceollin mixture	- Inhibit lipid peroxidation in liver, kidney, and brain tissue extracts	Kim et al. 2010 [82]
Central nervous system	Breast cancer MCF-7 cell line	Glyceollins mixture	- Increase nerve growth factor receptor gene expression	Zimmermann et al. 2010 [28]
	Glutamate-sensitive murine hippocampal HT22 cell line	Glyceollin mixture	- Attenuate glutamate-induced neurotoxicity - Suppress glutamate-induced intracellular ROS - Act through Nrf2 signaling pathway, activate hem oxygenase-1 enzyme	Seo et al. 2018 [85]
	Primary cortical neurons from wild-type and Nrf2 knockout male C57BL/6J mouse	Glyceollin mixture	- Suppress glutamate-induce cytotoxicity in primary cortical neurons of wild-type mice, but not cells from Nrf2 knockout.	Seo et al. 2018 [85]
	Wild-type and Nrf2 knockout male C57BL/6J mouse	Glyceollin mixture	-Improve cognitive deficits caused by scopolamine in wild-type mice, but no effect in Nrf2 knockout mice - Inhibit acetylcholine esterase activity in neurons from the cortex, but not in cells from the hippocampus	Seo et al. 2018 [85]
	Ovariectomized adult female CFW mouse	Glyceollin mixture	- Upregulate genes involved in neurogenesis, synaptic plasticity, tissue development and transcripts involved in signal transduction pathways, regulation of nerve impulse, cytoskeletal remodeling, and hormone signaling - Downregulate genes involved in neurodegeneration apoptosis and transcripts involved in developmental neurogenesis, synaptogenesis, and cell adhesion - Act through ER ^10^-dependent or ER-independent mechanisms depending on the target genes	Bamji et al. 2015 [86], Bamji et al. 2018 [87]
Inflammation	Murine macrophage RAW264.7 cell line	Glyceollin mixture	- Inhibit nitric oxide production and inflammatory mediators release through the NF-κB pathway	Kim et al. 2011 [88], Yoon et al. 2012 [89]
	Human umbilical vein endothelial cell line	Glyceollin mixture	- Reduce HMGB1 ^11^ protein level induced by LPS ^12^ - Decrease HMGB1-induced vascular barrier disruption - Decrease HMGB1-mediated pro-inflammatory and inflammatory response	Lee et al. 2014 [90]
	ICR mouse	Glyceollin mixture	- Reduce mouse ear swelling caused by 12-O-tetradecanoylphorbol-13-acetate	Kim et al. 2011 [88]
	Male C57BL/6 mouse that underwent cecal ligation and puncture	Glyceollin mixture	- Reduce serum HMGB1 level, decrease HMGB1-induced vascular barrier disruption - Reduce HMGB1-induced mortality	Lee et al. 2014 [90]
Cardiovascular diseases	Human aortic smooth muscle cell line	Glyceollin mixture	- Inhibit the cell proliferation and migration induced by PDGF-BB ^13^	Kim et al. 2012 [91]
	Aortic rings from male Sprague-Dawley rat	GI	- Reduce vascular contraction partly through the inhibition of the RhoA/Rho-kinase signaling pathway	Song et al. 2010 [92]
Melanogenesis	B16/B16F10 cell lines	Glyceollin mixture	- Inhibit melanin synthesis by decreasing melanogenic enzymes and other factors	Lee et al. 2010 [93], Shin et al. 2013 [94]
	Standard AB strain zebrafish (*Danio rerio*) embryos	Glyceollin mixture	- Decrease the pigmentation of the embryos, melanin synthesis and tyrosinase activity - Decrease *Sox10*, a pigment cell-specific gene	Shin et al. 2013 [94]

^1^ Glyceollin I, ^2^ Glyceollin II, ^3^ Glyceollin mixture contains glyceollin I, glyceollin II, and glyceollin III, ^4^ glucagon-like peptide 1, ^5^ glucose transporter 4, ^6^ very low-density lipoprotein, ^7^ low-density lipoprotein, ^8^ peroxisome proliferator-activated receptor, ^9^ reactive oxygen species, ^10^ estrogen receptor, ^11^ high mobility group box 1, ^12^ lipopolysaccharide, ^13^ platelet-derived growth factor BB.
